# Knockout of PERK protects rat Müller glial cells against OGD-induced endoplasmic reticulum stress-related apoptosis

**DOI:** 10.1186/s12886-023-03022-z

**Published:** 2023-06-23

**Authors:** Xiaorui Wang, Xinxing Zhu, Guangqian Huang, Lili Wu, Zhiyong Meng, Yuyu Wu

**Affiliations:** Department of Ophthalmology, Second Affiliated Hospital of Fujian Medical University, No.950, Donghai Street, Quanzhou, 362000 Fujian Province China

**Keywords:** Müller glia, Oxygen-glucose deprivation, Endoplasmic reticulum stress, Autophagy, Apoptosis

## Abstract

**Background:**

The pathological basis for many retinal diseases, retinal ischemia is also one of the most common causes of visual impairment. Numerous ocular diseases have been linked to Endoplasmic reticulum(ER)stress. However, there is still no clear understanding of the relationship between ER stress and Müller glial cells during retinal ischemia and hypoxia. This study examined the effects of ER stress on autophagy and apoptosis-related proteins, as well as the microtubule-related protein tau in rMC-1 cells.

**Methods:**

rMC-1 cells were cultured in vitro. RT-PCR、immunofluorescence and Western blotting revealed the expression levels of associated mRNAs and proteins, and the CCK-8 and flow cytometry assays detected cell apoptosis.

**Results:**

The results showed that under OGD(Oxygen-glucose deprivation) conditions, the number of rMC-1 cells was decreased, the PERK/eIF2a pathway was activated, and the expressions of p-tau, LC3、Beclin1 and Caspase-12 proteins were increased. After the PERK knockout, the expression of the above proteins was decreased, and the apoptosis was also decreased.

**Conclusion:**

According to the findings of this study, specific downregulation of PERK expression had an anti-apoptotic effect on OGD-conditioned rMC-1 cells. There is a possibility that this is one of the mechanisms of MG cell apoptosis during retinal ischemic injury.

**Supplementary Information:**

The online version contains supplementary material available at 10.1186/s12886-023-03022-z.

## Introduction

Endoplasmic reticulum (ER), as an important site for protein processing in organisms, can correctly fold and modify proteins [1]. When cells are stimulated by the outside world, the function of ER will also be damaged, and a large number of unfolded or misfolded proteins accumulate in ER and cannot be removed [2]. With ER stress induced, the unfolded protein response (UPR) is triggered. UPR plays an important role in alleviating ER stress by promoting the degradation of faulty proteins, decreasing protein synthesis, and enhancing protein folding ability. The UPR is primarily composed of three transmembrane proteins on the endoplasmic reticulum, namely PERK, IRE1 and ATF6. In the absence of external stimuli, three transmembrane proteins bind to GRP78. A persistent harmful stimulus causes GRP78 to dissociate from PERK, ATF6, and IRE1 and leads to ER stress. UPR activation is initially designed to alleviate ER stress resulting from misfolded proteins by suspending protein synthesis. However, when the endoplasmic reticulum stress time is too long or the UPR is insufficient to restore the endoplasmic reticulum homeostasis, the protein loss will affect the normal metabolism of the cell, which will result in the loss of biological function or the initiation of autophagy and apoptosis [3].

The autophagy process is a ubiquitous and highly conserved process of intracellular degradation in eukaryotes. Through autophagy, cytoplasmic components or damaged organelles will be degraded and released back into the cytoplasm for nutrient re-use. It helps the cell to resist the effects of adverse environmental conditions. The process of autophagy can be induced when cells are under the conditions of nutrient deficiency, low energy, and hypoxia. In this regard, autophagy serves as a primary mechanism of cytoprotection. Additionally, autophagy is also a mechanism for the death of cells [4], which is known as autophagic apoptosis (type II apoptosis). Whether autophagy promotes or inhibits cell death is still debated. In accordance with current research, autophagy must be maintained at an appropriate level. Insufficient or excessive autophagy will disrupt cellular homeostasis and cause disease. There is a highly conserved interaction between ER stress and autophagy in all types of cells, including yeast, mammals, and prokaryotes. Multiple researches have shown that [5–7], ER stress can induce autophagy in cells in a variety of ways. Mammalian cells can induce increased levels of intracellular autophagy by activating PERK, IRE1, or Ca2+.

Retinas are among the most metabolically active tissues in the body [8, 9].The retina converts light information received by the human eye into neuronal signals that can be processed by the brain. Since this process requires a great deal of energy, the retina is sensitive to changes in blood perfusion and oxygen concentration [10, 11]. A retinal ischemia and hypoxia occur when the blood circulation cannot meet the retina’s metabolic demands. It is widely believed that retinal ischemia and hypoxia contribute greatly to the pathogenesis of many retinal diseases, such as central retinal artery occlusions, central retinal vein occlusions, diabetic retinopathy, glaucoma, etc. [12–15]。There are a variety of inflammatory factors [16, 17] and neurotransmitters [18] that are expressed by ischemic retinopathy and contribute to retinal inflammation and retinal vascular dysfunction [19], as well as causing further insufficient blood flow and supply in the retina, as well as causing apoptosis of retinal ganglion cells and related retinal cells, leading to visual impairment and blindness [20].

It should be noted that insufficient circulation to the retina usually affects the inner retina and does not result in ischemia of the outer retina. Thus, retinal ischemia usually results in damage to neurons within the inner retina, while less damage is seen in photoreceptors [11]. In both humans and animals, retinal dot plots are shown [21, 22] that Hypoxia and ischemia primarily affect the b wave, which represents a mixed response between bipolar, amacrine, and Miller glia. B-wave amplitude decreases in relation to the severity and duration of the injury as well as the degree of recovery. Müller glia play an important role in supporting neurons and their functions, maintaining retinal metabolism, and maintaining the blood-retina barrier in the retina [23]. Whenever Müller glial cells appear to be dysfunctional, retinal neurons will degenerate [24].

As a component of neuronal fibrillary tangles, Tau has been extensively studied in Alzheimer’s disease and other neurodegenerative diseases. A recent study indicates that tau accumulation and phosphorylation play an important role in the pathogenesis of ischemic hypoxic retinal diseases [25, 26]. Additionally, activation of the UPR is closely associated with the accumulation and aggregation of p-tau in patients with Alzheimer’s disease and frontotemporal dementia [27, 28]. In the present study, we will examine the relationship between ER stress and autophagy, as well as the interaction between ER stress and Tau protein in Müller cells under ischemia and hypoxia. We hope to provide a new therapeutic target and a new approach to treating ischemic retinopathy.

## Materials and methods

### Primary cell culture and expansion

Rat Müller Glia cell(rMC-1) were bought from Beina Chuanglian Biotechnology Research Institute. rMC-1 cell line were cultures in Dulbecco’s Modified Eagle Medium: F-12 (DMEM/F12) supplemented with 1% penicillin/streptomycin (100U/mL), and 20% fetal bovine serum (Invitrogen-Gibco) at 5% CO2 and 37 °C. The culture medium is changed every 2 days, when the cells reach 80% confluence, cells were washed twice with PBS. The cells were digested with 0.25% pancreatin(Gibco trypsin -EDTA 0.25%) for 5 min. Then add bovine serum-containing medium to terminate digestion and replate through 1:2 dilutions.

### Oxygen-glucose deprivation (OGD)

The cells in the NC(negative control) group were treated without any other treatment. the cells in the OGD group and the transfection group were replaced with sugar-free and serum-free DMEM. Before starting the OGD, and N_2_ was used in a circulating three-gas incubator in advance to remove the residual O_2_, then put the cells into the incubator. The O_2_ of culture medium were continuously replaced by N_2_. The internal environment in the incubator (37 °C, 1%O_2_, 5% CO_2_, and 94%N_2_) was stable for 30 min, then the cells were culture for 12 h.

### RNA interference

The PERK siRNA-duplex was delivered using Lipo8000 RNAiMAX Transfection Reagent (ThermoFisher Scientific, USA) in Serum Medium. All transfections included a non-targeting control siRNA using 10 nM Silencer Select Negative Control No. 1 siRNA (Invitrogen, USA) and PERK transfection complexes were reverse transfected into Müller glia cells as per the manufacturer’s instructions. The Müller glia cells were incubated with transfection complexes for 48 h at 37 °C. Following incubation, cell lysates were collected for analysis or subjected to the desired treatment, after which cell lysates were collected for analysis of protein expression using western immunoblotting or flow cytometry.

the negative control: *UUCUCCGAACGUGUCACGTT*.

*ACGUGACACGUUCGGAGAATT*.

Rat si-PERK-655: *GGAUAGUGAUGAAAUGGAAGA*.

*UUCCAUUUCAUCACUAUCCCA*.

### Immunocytochemical staining

rMC-1 cells were plated in six-well plates. And the cells were processed for immunocytochemical staining with mouse glial fibrillary acidic protein (GFAP,80788T,CST), Glutamine synthetase (GS,ab176562,abcam). They were visualized with an inverted fluorescent microscope (Olympus, Tokyo, Japan).

### Immunofluorescence

rMC-1 cells were fixed at room temperature with 4% paraformaldehyde for 30 min and then washed with PBS. Permeabilize for 60 min with 0.1% BSA and 0.5% Triton X-100. The cells were blocked at room temperature with a 7.5% BSA blocking solution for 30 min. First antibodies LC3 (dilution 1:500, 12741T, CST) ,Beclin1 (dilution 1:500, 3495T,CST) and Caspase-12 (dilution 1:500, bs-1105R, Bioss) were then incubated overnight at 4 °C. The cells were subsequently incubated with fluorescent secondary antibody at 37 °C for 1 h and stained with PBST (phosphate buffer) wash nuclei, 0.001 mg/mL of DAPI(Thermo,USA) was added, and the cells were stored in the dark for 5 min.

### Detection of cell toxicity

rMC-1 cells were seeded into 96-well plates (1 × 10^4^ cells per well). Then, 24 h later,After 0 h、2 h、4 h、8 h、12 h, 10µL of cell counting kit 8 (CCK8, TAKARA, Tokyo, Japan) is added for 2 h. A spectrophotometer was ultimately used to measure the absorbance at 490 nm. An inhibition rate was determined to determine tunicamycin’s suppressive effect on HTMCs. We calculated the relative inhibition rate as a percentage as follows: (1-A experiment/A control) × 100%. Three independent experiments were conducted.

### Apoptosis assay

An apoptosis detection kit (BD Co., Ltd., Nanjing, China) was used for the assay. Cells were washed with PBS and resuspended gently in 1×Binding Buffer at a concentration of 1 × 106 cells/ml. Following this, pipette 100 ul solution (1*105 cells) into a 5 ml culture tube and add 5µL FITC annexin V and 5µL PI to the cell suspension. Add 400 µL of 1×Binding Buffer to each test tube and incubate at room temperature (25 °C) for 15 min in the dark. The apoptosis level of the cells in each group was determined using a FACS Calibur flow cytometer.

### RT-PCR

As mentioned in the previous paragraph, the cells were subjected to an assay for apoptosis. Afterwards, cells were lysed in TRI Reagent (Total RNA Isolation Reagent, Biosharp, CN), and stored at -80 °C. It was isolated according to the manufacturer’s instructions, which included treatment with chloroform, precipitation and recovery with isopropanol, washing with ethanol, and finally adding DEPC water to dissolve the RNA. Determine the concentration of the isolated RNA sample at 260 and 280 nm using an ultraviolet spectrophotometer, and evaluate the OD260/OD280 ratio to determine the purity of the sample. The purity is acceptable if the ratio is between 1.8 and 2.0. Reverse transcription of the RNA into cDNA was performed using the Reverse Transcriptase kit (TAKARA, Japan). Calculate the relative mRNA abundance by using the 2^(-ΔΔCt) method. Gene expression was normalized to GAPDH.The sequences of the primers were as follows, The primers were designed using NCBI.

GRP78-F: CGGTGTATTCAAGAACGGC.

GRP78-R : AAGGGTCATTCCAAGTGCG.

LC3-F: AACAACCAGGACAAGCAGG.

LC3-R: TCTCACCAGCATCGTAGAGG.

Tau-F: GTCCAAGTGTGGCTCAAAG.

Tau-R: GGGTGATGTTATCCAAGGAG.

GAPDH-F: ATCACCATCTTCCAGGAGC.

GAPDH-R: GGTTCACACCCATCACAAAC.

### Western blotting

Cells were treated with RIPA cell lysis buffer together with protease inhibitor and phosphorylase inhibitor and transferred to EP tubes which were placed on ice for 30 min. Cell lysis was performed using a centrifuge and centrifugation at 13,000 rpm for 5 min. Afterwards, the supernatant prepared for western blot analysis was transferred to another sterile EP tube. The membranes were blocked with 10% fat-free milk and incubated with the primary antibody selected from GRP78/BIP(ab108613, Abcam), PERK (5683t, CST), p-PERK (bs-3330R, Bioss),LC3 (12741T ,CST),Beclin1 (3495T,CST), GRP78/BIP (ab108613, Abcam), p-eif2α (5324T, CST), CHOP (2895T,CST), Tau (46687T,CST), p-Tau (20194T,CST), Caspase-12 (bs-1105R, Bioss)overnight at 4 C. Goat anti-rabbit IgG (1:5000, ab6721, Abcam, UK) was used as a secondary antibody.

### Statistical analysis

All experiments were conducted independently three times. All data were expressed as mean standard deviation (x̄ s) and analyzed using IBM SPSS 25.0 software. A normality test was conducted for each group of data. Those with uniform variance were tested using the group t-test or one-way analysis of variance, while those with non-uniform variance were tested using rank sum tests. With the test level α = 0.05, P < 0.05 indicated that the difference was statistically significant.

## Result

### Culture and identification of rat Müller glial cells

As described in the materials and methods, rMC-1 cells were cultured and identified successfully. Under an inverted microscope, the cultured cells can be observed the morphology of the cultured cells. Immunocytochemical staining revealed glial fibrillary acidic protein (GFAP, SFigure 1 A), laminin (G, SFigure 1B). Morphological and immunocytochemical results confirmed that the cells cultured in this study.

were Müller glial cells.

### OGD-induced rMC-1 hypoxia-ischemia cell model and promotes the expression of endoplasmic reticulum stress and autophagy-related proteins

**Fig. 1:**
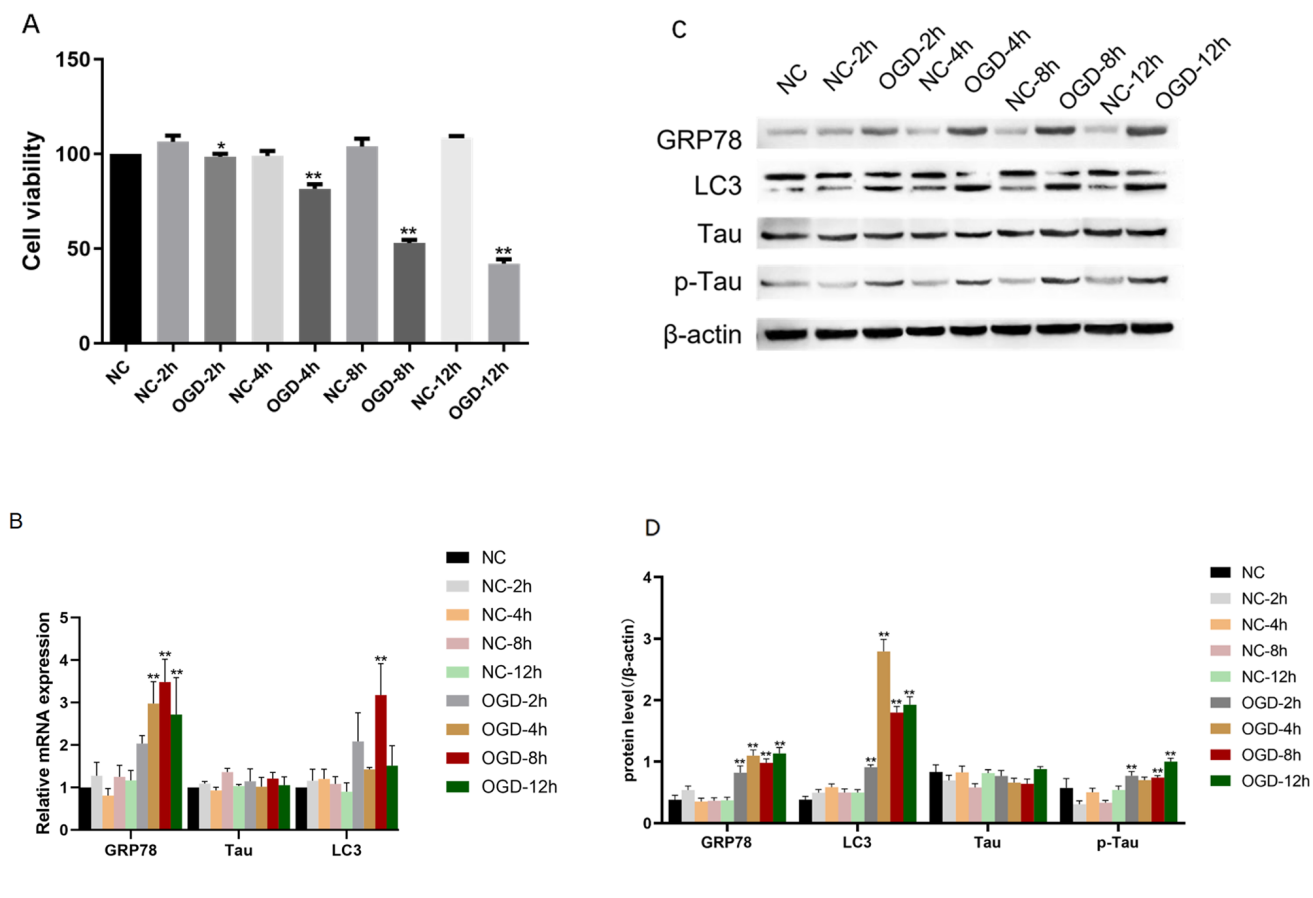
**OGD can induce MG injury and promote the expression of proteins related to endoplasmic reticulum stress and autophagy.** (A)CCK8 showed that OGD reduced the survival rate of MG, and the survival rate of MG was significantly decreased with the prolongation of OGD exposure. (B)RT-PCR was used to evaluate the effects of different durations of OGD exposure on the expression of GRP78, LC3, and tau(The relative mRNA expression was normalized by NC). (C) Representative blots. Strip was processed by Image J. (D) the histogram shows the protein densitometric analysis of GRP78, LC3, tau, and p-tau level. β-Actin was used as an internal control. Cells without any intervention are used as a negative control. Data were analyzed using group t-test. * P<0.05, ** P<0.01 (The OGD-4 h was compared to the NC-4 h,the OGD-8 h was compared to the NC-8 h, the OGD-12 h was compared to the NC-12 h).

To verify the success of the OGD-induced in rMC-1 hypoxia-ischemia cell model, we measured the viability of rMC-1 cells at different exposure times (2, 4, 8, and 12 h). CCK8 results showed that rMC-1 cells’ survival rates decreased with OGD exposure (Fig. 1a). The NC group and the OGD group were also compared in terms of protein expression levels of endoplasmic reticulum stress-related protein GRP78, autophagy-related protein LC3, misfolded protein Tau, and p-Tau at each time point. Results showed that GRP78, LC3, and p-Tau expression levels were significantly increased after exposure to OGD, but Tau expression levels were not significantly abnormal (Fig. 1b-d). Our results showed that OGD exposure at different times could induce time-dependent apoptosis of cells, so we used OGD 12 h for subsequent experiments.

### PERK-eIF2a-related signaling pathway involved in OGD-induced apoptosis of rMC-1 cells

**Fig. 2:**
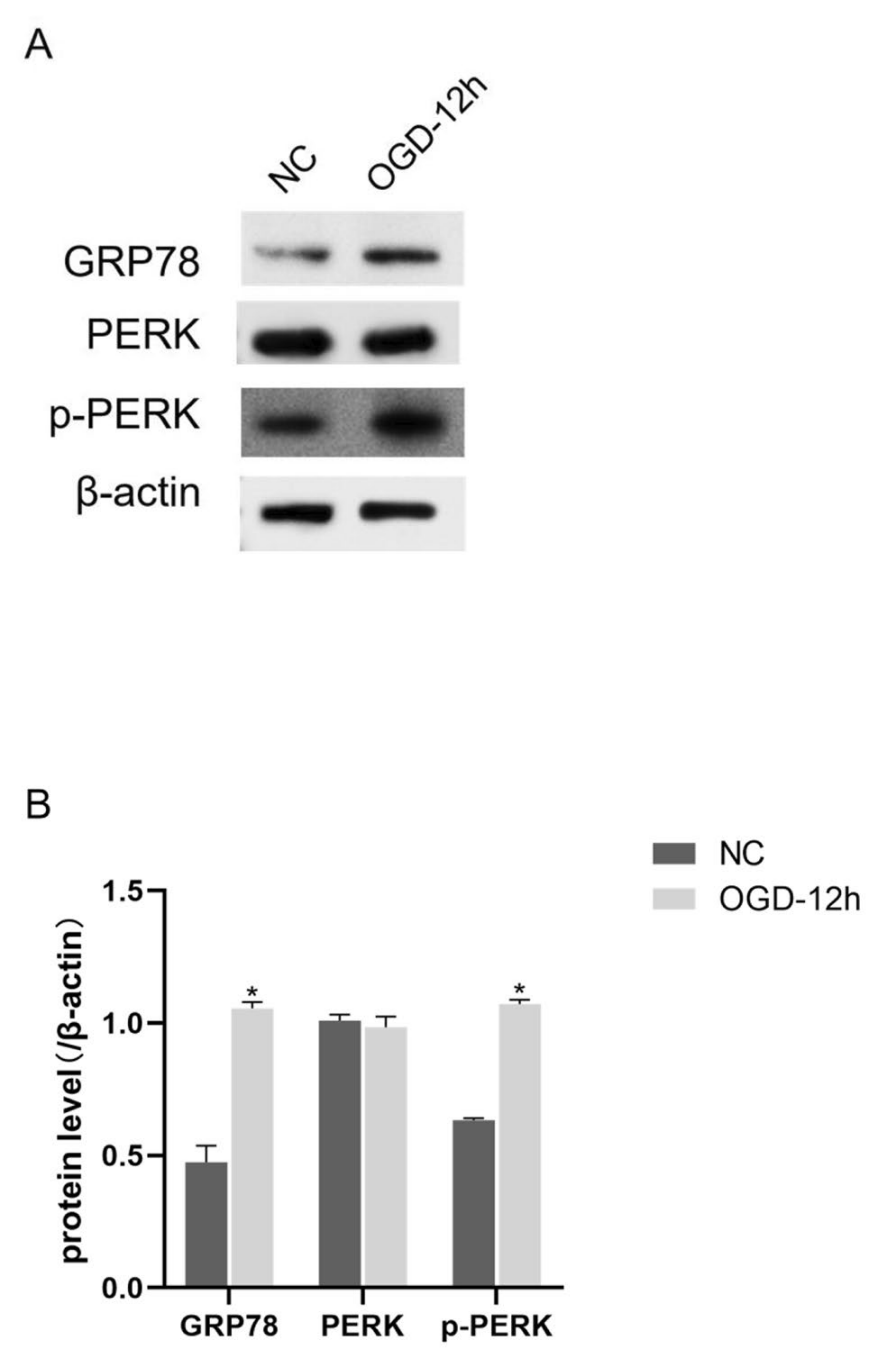
**Increased protein expression in the PERK/eIF2a pathway.** (A)Representative blots. Strip was processed by Image J. (B)the histogram shows the Densitometry of GRP78、PERK and p-PERK protein signals. β-Actin was used as an internal control. Data were analyzed using group t-test. * P<0.05, **P<0.01 (The OGD-12 h was compared to the NC).

In order to investigate the role of endoplasmic reticulum stress in OGD-induced apoptosis, we compared the expression levels of endoplasmic reticulum stress-related proteins between the NC group and the OGD12h group by Western blot (Fig. 2). As compared with the NC group, the OGD 12 h exposure group showed increased levels of p-PERK, eIF2a and p-eIF2a protein expression, while PERK protein expression was not significantly affected. Based on these results, we speculated that the PERK-eIF2a-related signaling pathway in endoplasmic reticulum stress might be involved in the process of apoptosis.

### Knock-out of PERK affects autophagy and apoptosis-related proteins, as well as expression of p-tau in rMC-1 cells

**Fig. 3:**
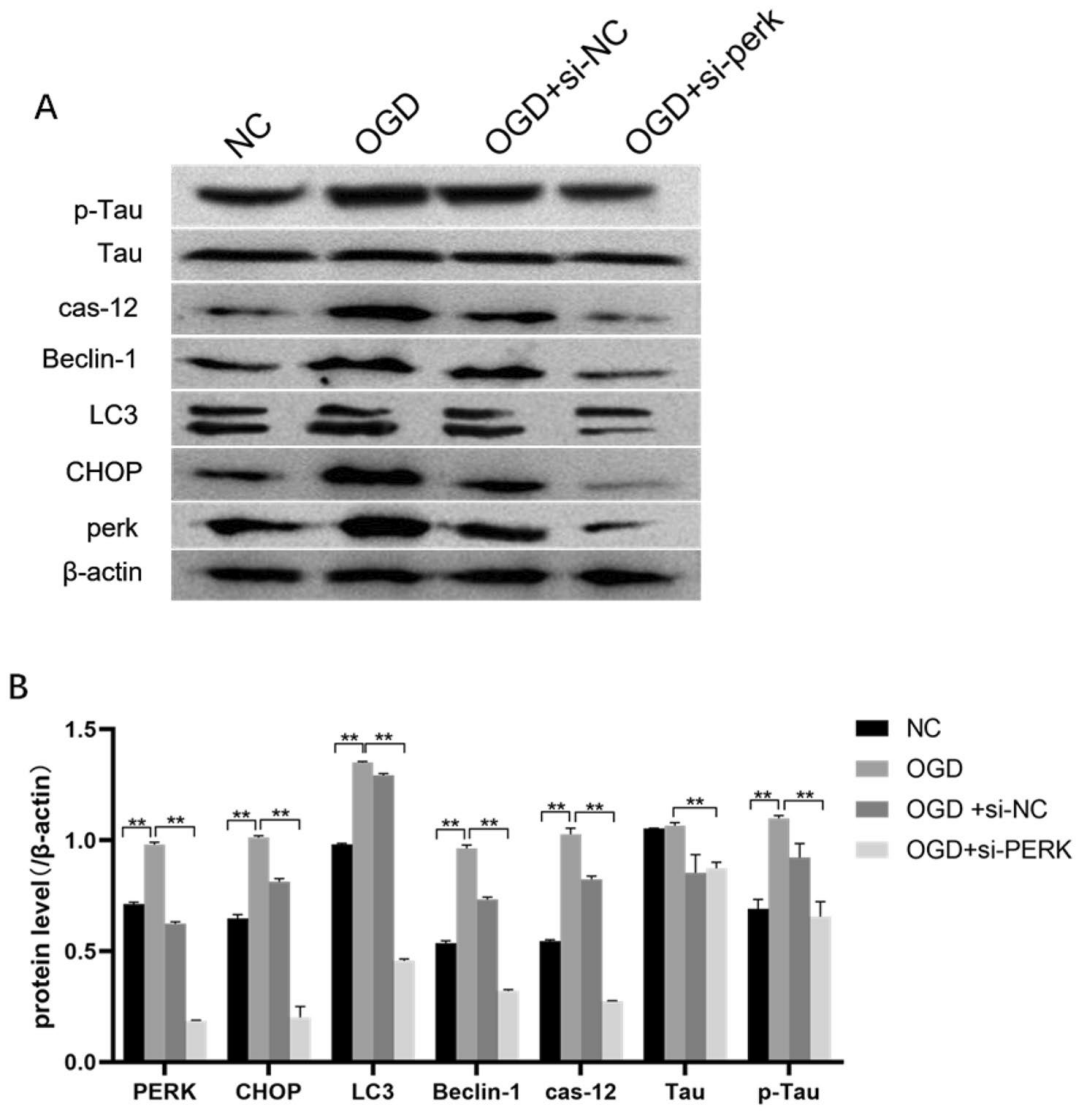
**The Effects of knock down of PERK on the expressions of autophagy and apoptosis-related proteins on MG cell.** (A) Representative blots. Strip was processed by Image J. (B)The histogram shows analysis of each group cell apoptosis related proteins’ expression. Data were analyzed using group t-test. * P<0.05, **P<0.01

In order to confirm the role of PERK in OGD-induced apoptosis of rMC-1 cells, si-PERK was transfected into rMC-1 cells under OGD conditions, and the protein expression level of PERK was decreased after transfection, indicating that si-PERK transfection was successful, To further verify the relationship between PERK and intracellular autophagy and apoptosis in rMC-1 cells under the conditions of OGD, we measured the expressions of autophagy and apoptosis-related proteins by western blot (Fig. 3). The results showed that the increased autophagy-related proteins LC3 and Beclin-1 as well as the apoptosis-related proteins CHOP and caspase-12 in the environment exposed to OGD could be down-regulated by silenced PERK, thus indicating that the expression levels of these proteins could be reduced by suppressing PERK. Furthermore, we measured the expression levels of the microtubule-associated proteins Tau and p-Tau, finding that PERK decreased p-Tau expression levels when its expression was reduced.

### The effect of knockout PERK on autophagy and apoptosis in rMC-1 cells was detected by immunofluorescence

**Fig. 4:**
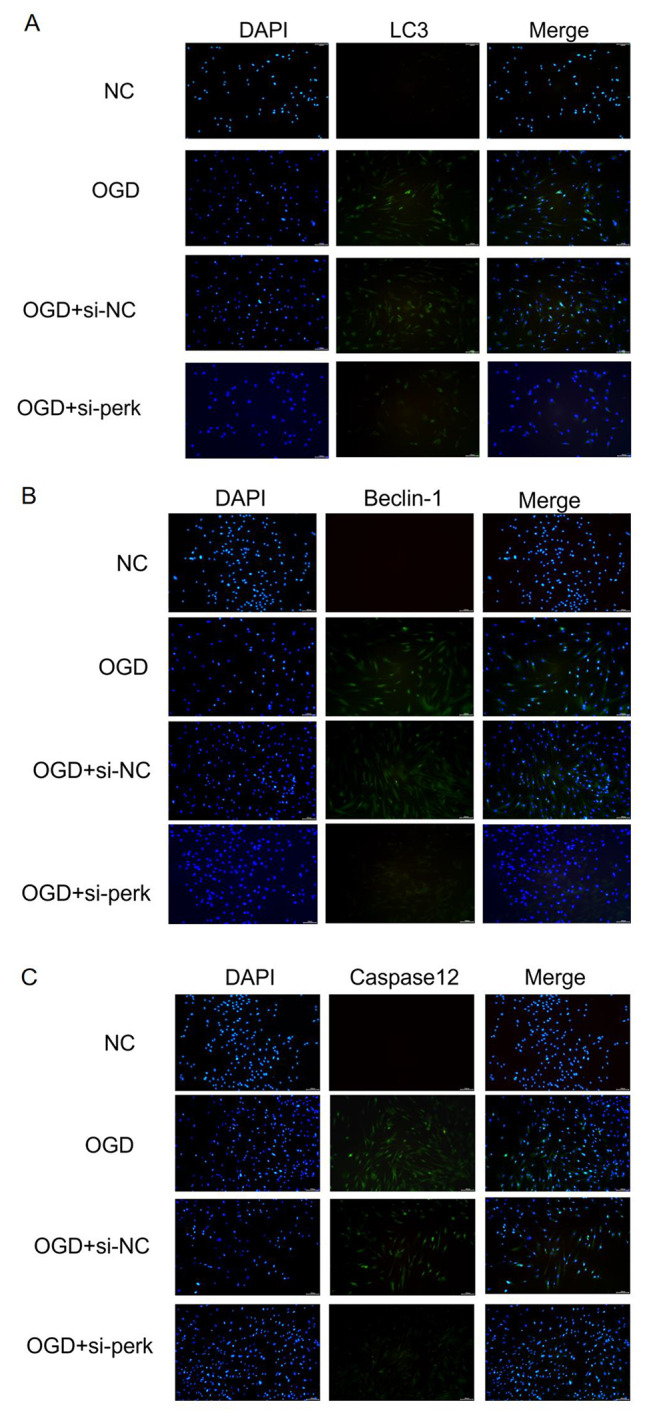
**Silencing of PERK inhibits OGD-induced autophagy and apoptosis in rMC-1 cells.** (A-C) Representative images stained with LC3, Belcin1, and Caspase-12 in sequence. Scale bar: 100 μm

Through an immunofluorescence experiment(Fig. 4), we found that PERK plays a role in intracellular autophagy induced by OGD. Compared to the NC group, the OGD group showed significantly higher fluorescence levels for autophagy-related proteins such as Beclin-1 and LC3 and the apoptosis-related protein Caspase12, while the fluorescence levels of the above proteins in the OGD group were significantly lower than those in the OGD + si-PERK group. The results showed that OGD exposure could increase the expression of autophagy-related proteins Beclin-1 and LC3 and the apoptosis-related protein Caspase12 in rMC-1 cells, while targeted down-regulation of PERK could reduce the expression of these proteins.

### Knock-out of PERK alleviates OGD-induced apoptosis in rMC-1 cells

**Fig. 5:**
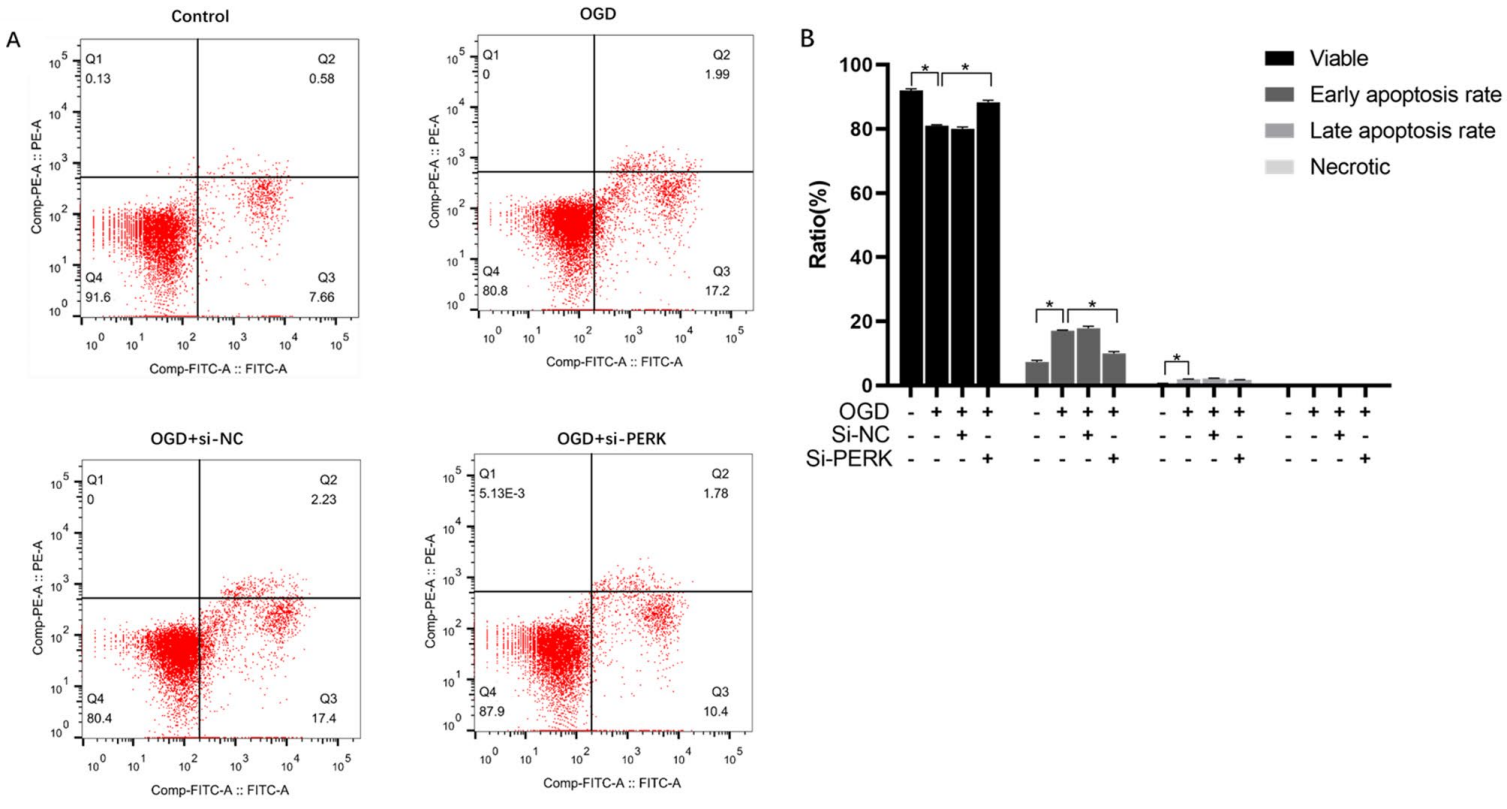
**Effects of knock down of PERK overexpression on MG cell apoptosis ratio.** (A) The ratios of viable, early apoptosis, late apoptosis, and necrotic cells detected by Flow cytometry. (B)the histogram shows analysis of each group cell apoptosis ratio. * P<0.05

In order to confirm the role of PERK in OGD-induced apoptosis of rMC-1 cells, We detected the apoptosis rate of cells in each group by flow cytometry (Fig. 5). the OGD group had a 10.8% decrease in surviving cells compared with NC(p<0.05). The proportion of early apoptotic cells increased by 9.54%, while the proportion of late apoptotic cells decreased by 1.41%, suggesting that OGD may promote apoptosis. Compared with the OGD group, the OGD + siPERK group showed a 7.1% increase in surviving cells (p<0.05), 6.8% decrease in early apoptotic cells, and 0.21% decrease in late apoptotic cells. Based on these findings, it might be possible to resist OGD-induced apoptosis by reducing PERK expression in rMC-1 cells.

## Discussion

Our main objective in this study was to determine the effects of ER stress induced by exposure to simulated ischemia and hypoxia on autophagy and tau protein in vitro. According to our results, specific reduction of PERK expression in endoplasmic reticulum stress molecules was shown to reduce autophagy and p-tau expression at the same time and improve rMC-1 cell survival. This study provides evidence supporting the role of PERK/eIF2α in ischemic hypoxic retinal disease.

Until recently, most studies on Müller glial cells have focused on the activation of glia and functional changes of Müller glial cells in various pathological environments. rMC-1 cells in the OGD environment have not been studied for endoplasmic reticulum stress and apoptosis. Compared with retinal neurons that are highly sensitive to insufficient blood supply, Müller glial cells have a significant resistance to ischemia and hypoxia or hypoglycemia, resulting in less damage and apoptosis of Müller glial cells [29, 30].Perhaps this is due to Müller glial cells’ special energy metabolism [31]. However, this does not imply that there is no significance to the decrease in Müller glial cells. As the most abundant glial cells in the retina, Müller glial cells contribute to neuronal development, retinal metabolism, and neurotransmitter transmission [32, 33]. Additionally, the function and survival of Müller glial cells are essential for the survival of RGCs [34]. Therefore, We believe it is also crucial to reduce Müller glial cells apoptosis in the presence of retinal ischemia and hypoxia.

According to Figs. 1 and 2, under the condition of OGD, rMC-1 cells were reduced, PERK and eIF2a phosphorylation levels were increased, and autophagy-related proteins were expressed. As a transmembrane protein on the endoplasmic reticulum, PERK is activated by overload of excessive misfolded protein in the endoplasmic reticulum and subsequently phosphorylates eukaryotic translation initiation factor 2-α [35],thereby stopping protein synthesis and cellular metabolic function so as to have time to reduce stress caused by misfolded protein. However, eIF2α remained phosphorylated due to prolonged cellular stress, thereby blocking trophic factor mRNA translation and protein synthesis for an extended period of time. This leads to the loss of cell function or death due to the lack of important nutritional support [36].Moreover, persistence of ER stress can activate the CHOP secondary pro-apoptotic pathway, ultimately leading to cell death [37]. As the CHOP pathway is activated, it not only reduces the expression of the anti-apoptotic Bcl-2 protein family, but also increases the expression of apoptotic proteins such as Caspase-12 on endoplasmic reticulum [37].

To verify PERK’s effects on autophagy and apoptosis, we performed a specific knockout experiment in cells.

As shown in the Figs. 3, 4 and 5, after the PERK was knocked out under the OGD condition, the expressions of LC3 II/LC3 I, Belcin1, Chop, and Caspase-12 were reduced and cell viability was increased. As mentioned above, the PERK/eIF2α signaling pathway plays an integral role in endoplasmic reticulum stress. As a result of the prolonged cellular stress, eIF2a remains phosphorylated, preventing mRNA translation for trophic factors and protein synthesis for a long time. There is a loss of function or death of cells as a result of the lack of nutritional support. Previous studies have shown that the activation of the PERK/eIF2α pathway is crucial for maintaining autophagy in cells [38, 39]。Autophagy, as a means for cells to maintain steady state and resist environmental pressure, is an important mechanism for cell survival [40]。However, excessive autophagy will cause cell apoptosis. Some studies have shown that autophagy plays an influential role in the apoptosis of Müller glial cells and RGCs induced by retinal ischemic disease [41, 42].Based on our previous research [43], we demonstrated that LC3 and Beclin1 were involved in the apoptosis of RGCs in a model of acute retinal ischemia-reperfusion in rats. The above results have shown that under the conditions of OGD, the PERK/eIF2α pathway was involved in the apoptosis of rMC-1 cell, with the activation of PERK being able to up-regulate the expressions of autophagy-related proteins LC3 and Beclin1, as well as the apoptotic proteins Chop and Caspase-12.

In Figs. 1 and 3 we found that p-Tau expression was upregulated in rMC-1 cells under the OGD condition, but it was decreased after the specific knockout of PERK, indicating that tau and PERK may interact. Tau protein, as the only essential component of neurofibrillary tangles, is widely distributed in the neurons of the central nervous system. Its main physiological function is to stabilize the microtubule network in the neuron by combining with microtubules. When multiple sites are phosphorylated, it will cause damage to neuronal cell transportation, synapses,and mitochondrial integrity [44].Previous studies have shown that hyperphosphorylation of tau protein was also considered to be the basis for many neurodegenerative diseases in previous studies [45], There has been previous evidence that the accumulation and activation of tau in cells can activate the PERK-related endoplasmic reticulum stress pathway [46].In recent studies, increased expression of p-tau in the retina has been detected in several ischemic retinal diseases (such as diabetic retinopathy and glaucoma). Researchers have found that hyperphosphorylation of tau in diabetic rodent retinas induces retinal neurodegeneration by destroying synaptic and mitochondrial functions [47].Rodent models with high intraocular pressure can benefit from intraocular injections that reduce phosphorylated tau levels [48].This indicates that phosphorylation of tau protein in the intraocular retina can be toxic to RGC. PERK inhibitors have been shown in recent studies to reduce tau phosphorylation in the mouse brain and prevent tau-induced neurodegeneration. PERK was reported to be able to inhibit tau phosphorylation by Radford et al., as it reduced the expression level of PERK and at the same time reduced the inhibitory function of eIF2a in the translation of proteins [49].

Studies have demonstrated the importance of endoplasmic reticulum stress and autophagy in ischemic eye disease. There is still no clear understanding of how endoplasmic reticulum stress regulates autophagy and tau protein. This study aimed to determine whether endoplasmic reticulum stress-related autophagy induced by simulated ischemia and hypoxia affected proliferation and apoptosis in Müller glial cells cells in vitro, and to test the effectiveness of the knock-out effects on Müller glial cells apoptosis after the related endoplasmic reticulum stress-related molecules were knocked out. According to our knowledge, this is the first study of its kind. We demonstrated that rMC-1 cell survival was improved during hypoxia and ischemia by reducing the expression of PERK. The PEKR/eIF2a signaling pathway is involved in the apoptosis of rMC-1 cells caused by ischemia and hypoxia, and it can activate the intracellular autophagy pathway. By inhibiting PERK expression specifically, autophagy-related proteins and endoplasmic reticulum-related apoptotic proteins were decreased in cells, and tau phosphorylation was decreased. While cell survival increases.It was hypothesized that when cells were stimulated by external stress, misfolded intracellular proteins accumulated, and tau could activate the PERK/eIF2α signaling pathway of endoplasmic reticulum stress. PERK, as an important target capable of inhibiting endoplasmic reticulum stress, could reduce the phosphorylation level of tau. The results of this study are similar to those of previous studies [49, 50], whereas in some studies, PERK activators protected cells from damage after tau phosphorylation [36, 51]。.

However, The ATF6 and IRE1 signaling pathways were not investigated in this study, nor were the specific effects of elevated tau phosphorylation on rMC-1 cell sinvestigated. We will supplement this in subsequent studies.

To conclude, we have demonstrated that the PERK/eIF2a signaling pathway is involved in the induction of apoptosis in Müller glial cells under the conditions of OGD. Additionally, we demonstrated that activation of PERK also results in tau phosphorylation in rMC-1 cells under the conditions of OGD. Higher levels of tau phosphorylation can damage glial cells, leading to dysfunction and degeneration of nerve cells. Considering the importance of retinal ischemia and hypoxia in retinopathies, our research results provide increasingly compelling evidence for Optic nerve ischemia and hypoxia therapy. These therapies aim to reduce autophagy and decrease tau protein phosphorylation, and protect Müller glial cells from damage to maintain the retinal neuron population.

## Electronic supplementary material

Below is the link to the electronic supplementary material.

Supplementary Material 1

Supplementary Material 2

## Declarations

### Availability of data and materials

All data relevant to this study is available upon reasonable request from the corresponding author.
