# Supplementary material for: Knockout of PERK protects rat Müller glial cells against OGD-induced endoplasmic reticulum stress-related apoptosis
Source: BMC Ophthalmol. 2023 Jun 23;23:286. doi: 10.1186/s12886-023-03022-z (PMC10290337; doi:10.1186/s12886-023-03022-z)
Supplement: Supplementary file 1 — Supplementary Material 1 [file 12886_2023_3022_MOESM1_ESM.docx]

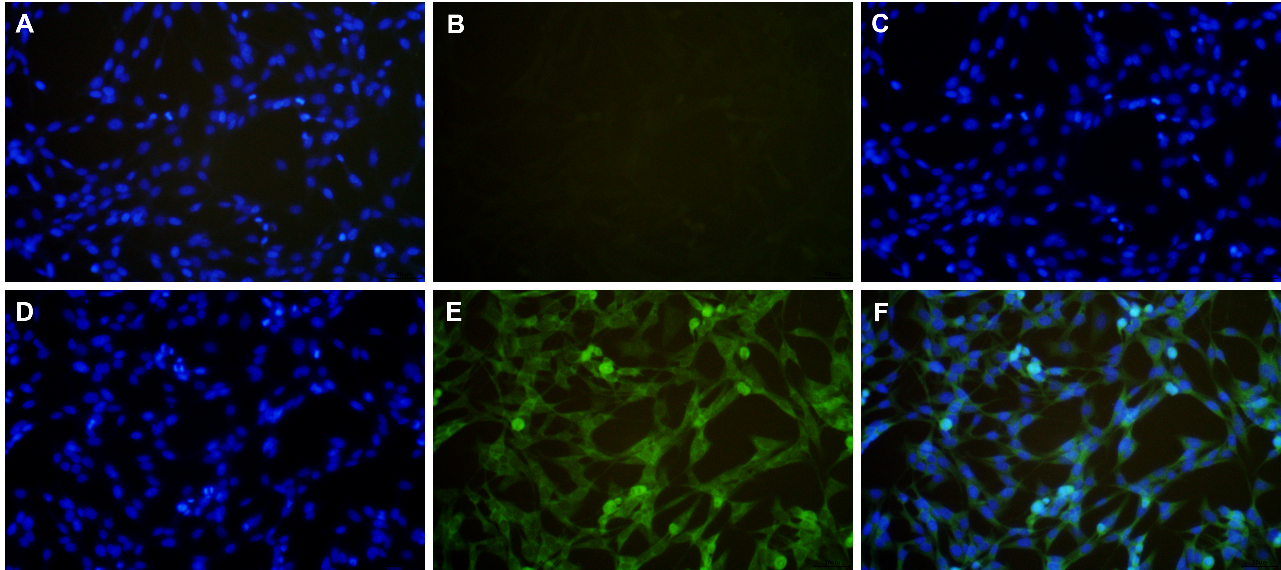


SFigure 1. Identification of rMC-1 cells (100×). **A-C**: Glial fibrillary acidic protein (GFAP) staining. **D-F**: Glutamine synthetase (GS) staining
