# Supplementary material for: Knockout of PERK protects rat Müller glial cells against OGD-induced endoplasmic reticulum stress-related apoptosis
Source: BMC Ophthalmol. 2023 Jun 23;23:286. doi: 10.1186/s12886-023-03022-z (PMC10290337; doi:10.1186/s12886-023-03022-z)
Supplement: Supplementary file 2 — Supplementary Material 2 [file 12886_2023_3022_MOESM2_ESM.docx]

Figure1C GRP78


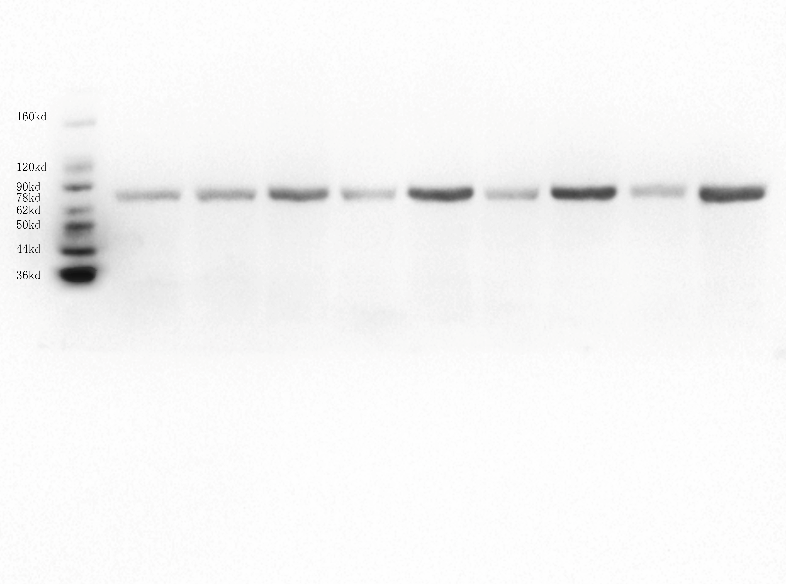


Figure1C LC3


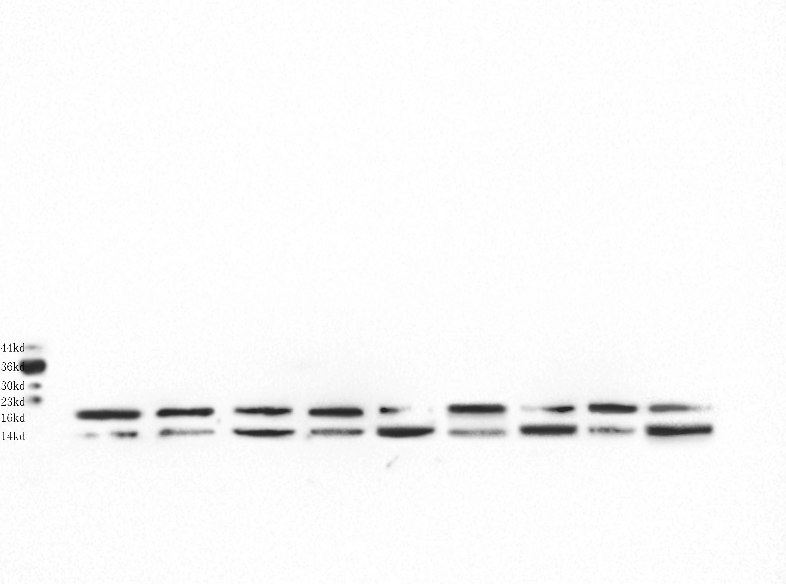


Figure1C p-Tau


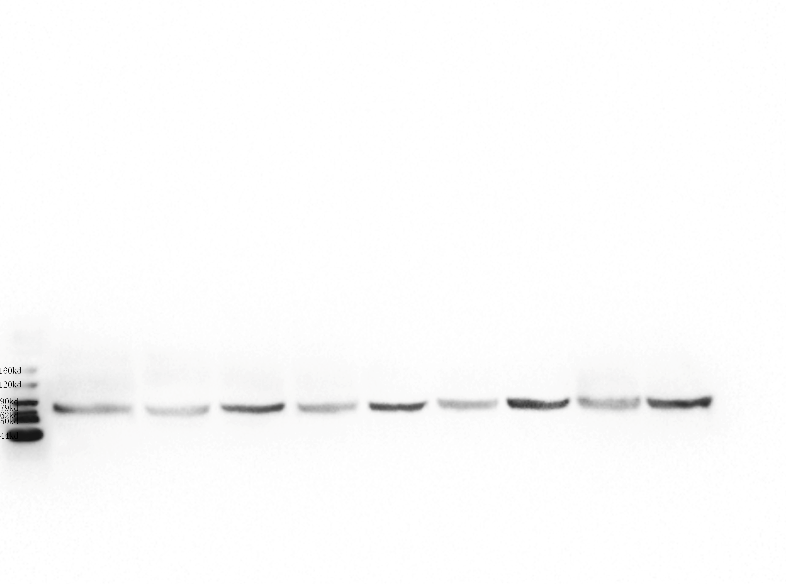


Figure 1C Tau


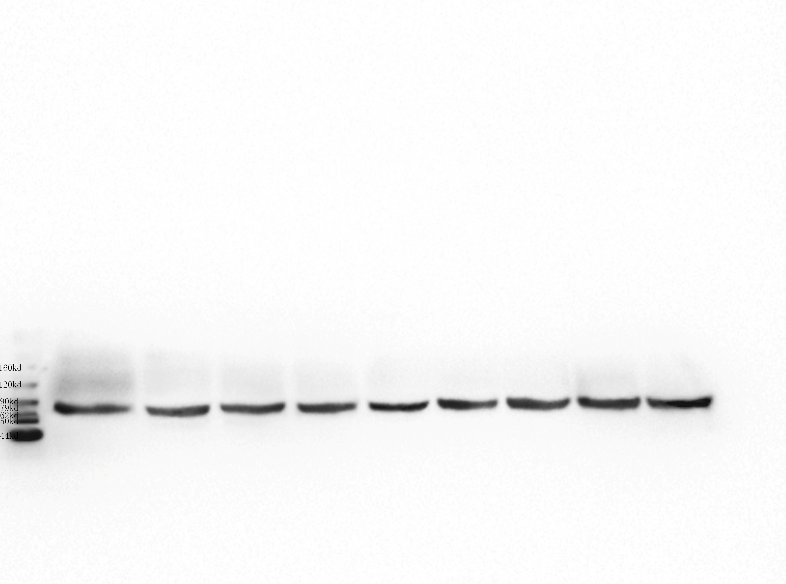


Figure1C β-actin


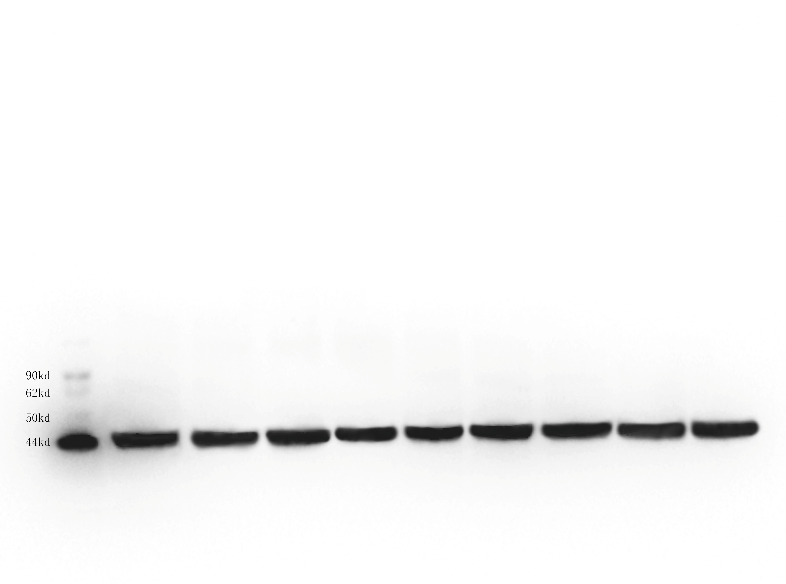


Figure2A GRP78





Figure2A PERK





Figure2A p-PERK


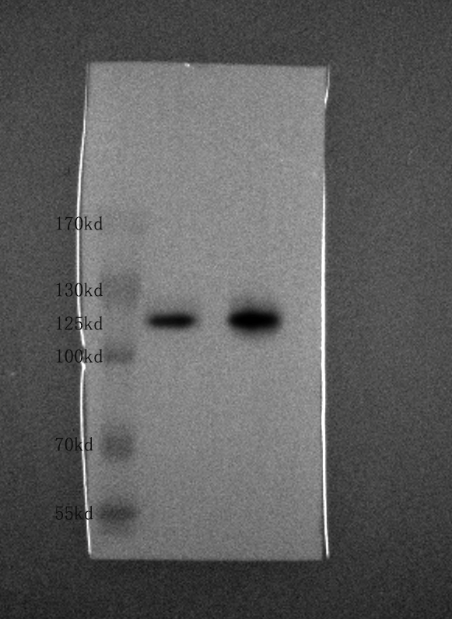


Figure2A β-actin





Figure3A cas-12





Figure3A CHOP





Figure3A LC3





Figure3A perk





Figure3A Tau


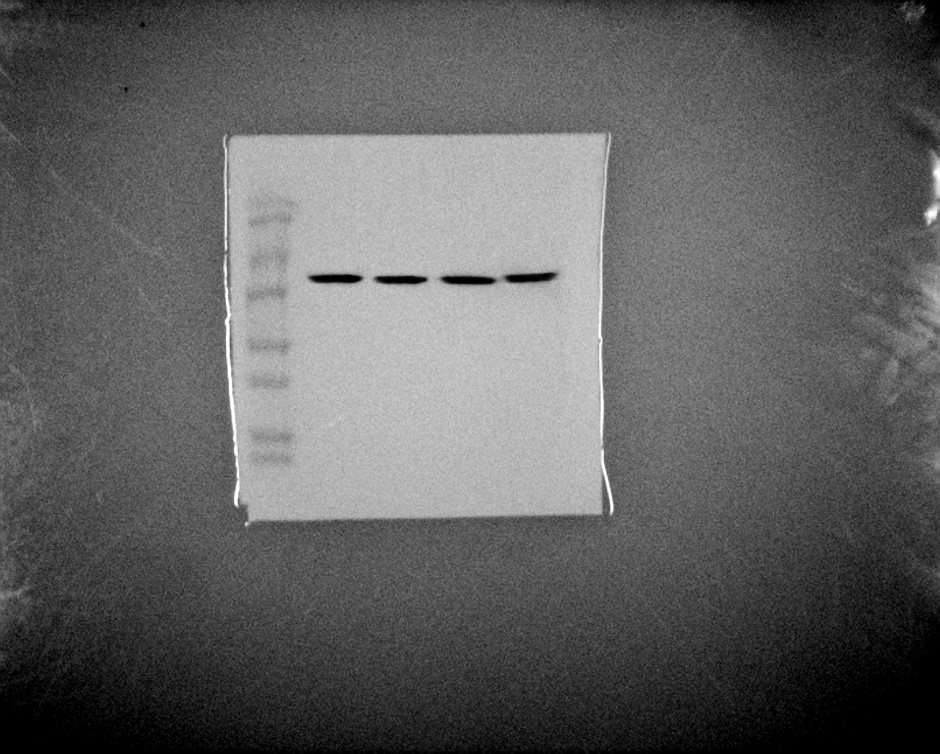


Figure3A p-Tau





Figure3A β-actin





Figure3A Beclin-1
